# The study protocol of the evaluation for the preventive efficacy of the HPV vaccine for persistent HPV16/18 infection in Japanese adult women: the HAKUOH study

**DOI:** 10.1186/s12885-020-07563-0

**Published:** 2020-11-03

**Authors:** Tetsuji Kurokawa, Makoto Yamamoto, Toshimichi Onuma, Hideaki Tsuyoshi, Akiko Shinagawa, Yoko Chino, Yoshio Yoshida

**Affiliations:** Department of Gynecology and Obstetrics, Faculty of Medical Sciences, University of Fukui, 23-3 Matsuoka, Shimoaizuki, Eiheiji-cho, Yoshida-gun, Fukui, 910-1193 Japan

**Keywords:** Human papillomavirus, Vaccine, Cervical cancer, Catch-up program

## Abstract

**Background:**

In general, human papillomavirus (HPV) vaccines have demonstrated efficacy in young women worldwide, but there is limited evidence on the efficacy of the quadrivalent HPV6/11/16/18 vaccine in adult women and no evidence of its effectiveness in Japanese adult women in particular. This study aims to evaluate the efficacy of the quadrivalent HPV6/11/16/18 vaccine for persistent HPV16/18 infection in Japanese women aged 27–45 years.

**Methods:**

This is an interventional, nonrandomized, non-double-blind prospective cohort study designed to compare the rates of persistent HPV16/18 infection between the vaccinated arm and unvaccinated arm. The subjects will consist of all women aged 27–45 years who have normal cytology results confirmed by cervical cancer screening from May 2019 to March 2021. The follow-up time is two years. The subjects will be divided into two groups: the vaccinated group and the unvaccinated group. The study will need to enroll 600 vaccinated participants (experimental arm) and 2200 unvaccinated participants (control arm).

**Discussion:**

The findings of this trial (HAKUOH study) might provide the first local evidence on the subject and be significantly useful not only to medical academia but also to the Japanese Ministry of Health, Labour and Welfare. The findings could contribute to public health improvement by providing local supportive knowledge on the prevention of HPV infection through HPV vaccination in young adult women in Japan, where active recommendations have been suspended for a long time due to adverse effects.

**Trial registration:**

Trial registration number: NCT04022148.

Registration began on December 1, 2019.

## Background

In April 2013, the Ministry of Health, Labour and Welfare (MHLW) started a national vaccination program for women aged 12–16 years in Japan. However, human papillomavirus (HPV) vaccinations were reportedly associated with serious adverse events, and in June 2013, the MHLW announced the suspension of the active recommendation of HPV vaccination. Since then, the vaccination rate in Japan has declined drastically from approximately 70% in 2013 to 1% in 2018 [1].

The very low vaccination rate in Japan has attracted great attention among different institutions. The Japanese Society of Obstetrics and Gynecology, Japanese Society of Gynecologic Oncology, and Japanese Association of Obstetricians and Gynecologists have advised the MHLW to re-implement their strong recommendation for HPV vaccination. The Global Advisory Committee on Vaccine Safety also stated no reason to suspend HPV vaccination or its active recommendation. However, their advice has not led to the MHLW reversing their decision to suspend the active recommendation [2, 3], and a longer time is expected before a strong recommendation is made again.

A previous study showed that the HPV infection rate will increase for every 1-year delay in resuming the active recommendation [4]. This finding suggests that the age of the high-infection-rate group will also increase. Globally, there is limited evidence on the effectiveness of the HPV vaccine in adult women [5, 6], and to our knowledge, there are no available data on the effectiveness of the vaccine in Japanese adult women. Therefore, data on the effectiveness of the vaccine in Japanese adult women will be very valuable and are urgently needed for the catch-up program for these women, as they were not vaccinated for HPV after the active recommendation for vaccination was suspended.

Adult women are not covered under the national immunization program of Japan due to cost-effectiveness concerns, which might be attributed to the high prevalence of persistent HPV16/18 infection in this population. In this study, we will focus on women with normal cytology results because we verified that approximately 99% of the women with normal cytology results have HPV16/18 negative results, as described in our previous study (Fukui Cervical Screening [FCCS] study) [7]. This study (the HAKUOH study) therefore aims to evaluate the efficacy of the quadrivalent HPV6/11/16/18 vaccine (Gardasil®) in healthy Japanese young adult women (aged 27–45 years) with normal cytology results on cervical cancer screening.

## Methods

### Study design

**Fig. 1:**
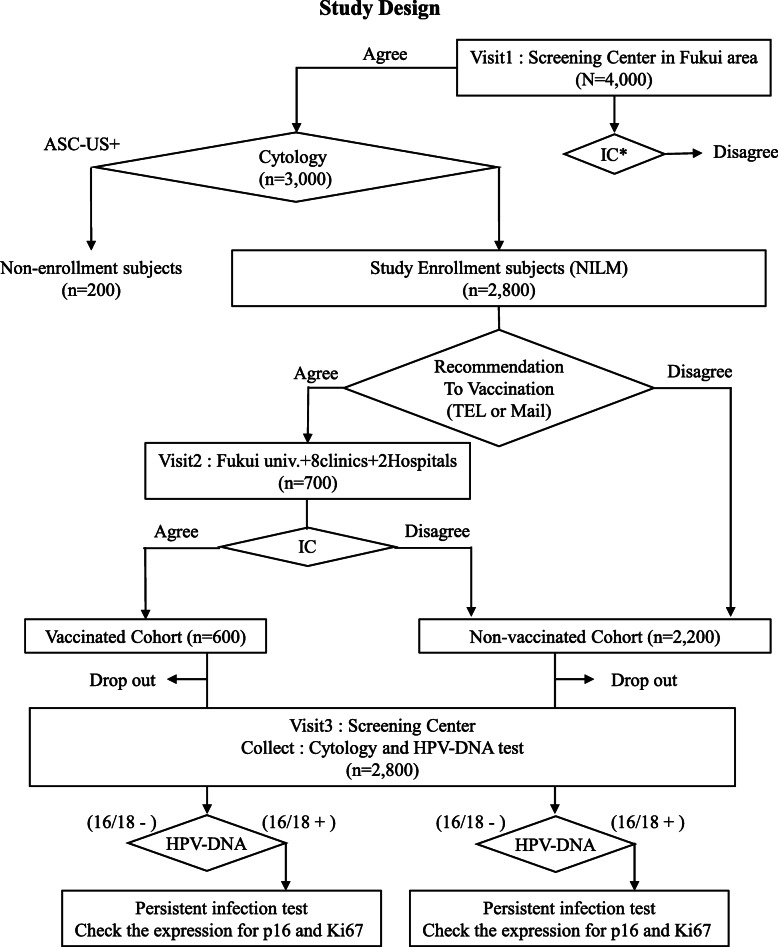
Study design. IC*: IC to participant in the study and to be sent the recommendation for vaccination

This is an interventional, nonrandomized, non-double-blind prospective cohort study that aims to compare the rate of persistent HPV16/18 infection between the vaccinated arm and unvaccinated arm, as shown in Fig. 1.

### Subjects and vaccine

The subjects will consist of all women aged 27–45 years who have normal cytology results confirmed by cervical cancer screening programs from May 2019 to March 2021. All women will have Japanese nationality and live in the Fukui Prefecture of Japan. In the Japanese cervical cancer screening program, the government covers the cost of cytology tests. Women are screened every 2 years. The uptake rate for cervical cancer screening in Japan is approximately 40%, with no regional differences. The data collection began in December 2019. The follow-up time will be 2 years because in the Japanese cervical cancer screening program, women are screened every 2 years. The inclusion criteria are as follows: normal cytology results confirmed by cervical cancer screening from May 2019 to March 2020, aged 27–45 years, an intact uterus, and willingness to undergo the HPV-DNA test (cobas4800) within 12 months. The exclusion criteria are as follows: pregnancy, treatment or follow-up evaluation for CIN within the previous 12 months, and previous administration of the HPV vaccine. The subjects will complete the questionnaire about sexual behavior and whether or not they were previously tested for HPV. The subjects will be divided into two groups: the vaccinated group and the unvaccinated group. In the experimental arm comprises women who have been vaccinated three times in 1 year after NILM confirmation by cytology. These women will participate in the cervical cancer screening program after 2 years. We will obtain a residual part of the sample for cytology, and the sample will be used for the HPV test. In women with HPV16/18-positive cells, p16 and Ki67 expression will be analyzed to judge persistent infection. The control arm will comprise women who participate only in the cervical cancer screening program after 2 years. We will obtain a residual part of the sample for cytology, and the sample will be used for the HPV test. In women with HPV16/18-positive cells, p16 and Ki67 expression will be analyzed to judge persistent infection. Subjects in 11 hospitals/clinics who consent to be vaccinated will be enrolled in the vaccinated group and will receive the quadrivalent HPV6/11/16/18 vaccine (Gardasil®) at day 1, month 2, and month 6. Subjects who refuse to be vaccinated will be enrolled in the unvaccinated group.

### Efficacy end point and case definition

The primary efficacy end point is the incidence of persistent HPV16/18-related infection over 2 years. The secondary efficacy end point is twofold: the incidence of persistent HPV16/18-related infection over 2 years by age group (27–30/31–35/36–40/41–45 years) and the consent rate for vaccination by age group (27–30/31–35/36–40/41–45 years). Women in both arms will receive routine cytology screening and an HPV-DNA test (cobas® 4800 system; Roche Diagnostics, Mannheim, Germany) 2 years after the first visit. Among women with HPV16/18-positive cells in both groups, p16 and Ki67 expression will be analyzed using the CINtec® PLUS kit (Roche Diagnostics). Persistent infection is defined as cases in which there are HPV16/18-positive cells overexpressing p16 and Ki67 [8].

### Statistical analysis

Data for HPV16/18 infection, cervical lesions and potential confounders (e.g., age, sexual activity status) from all subjects will be included in the analysis. The association among all data will be analyzed. The chi-squared test will be used to compare the incidence of persistent infection between the vaccinated and unvaccinated groups. *P*-values of < 0.05 will be considered statistically significant. All statistical analyses will be carried out using SPSS version 25.

### Sample size justification

Based on the assumed incidence rate of persistent infection among adult women (0.9 per 100 person-years) and an assumed risk reduction of 80% [8], for a two-sided test of the null hypothesis, the ideal sample size of the vaccinated group is 537 women and that of the unvaccinated group is 1611 women [9]. A power of 80% and a significance level of 0.05 will be set to control for a type I error. We discussed the sample size justification during the research meeting among researchers. Assuming that the dropout rate is 10% in the vaccinated group and 25% in the unvaccinated group, we will need approximately 600 vaccinated women and 2200 unvaccinated women. To enroll 600 vaccinated participants, it will be necessary to obtain informed consent from 700 participants, assuming that 85% of women actually get inoculated after receiving explanations of the vaccine and agreeing to be inoculated. Additionally, to enroll approximately 2200 unvaccinated participants, 2100 women who do not consent for vaccination will be included in the group in addition to the 15% of women mentioned above who will not actually get inoculated. Therefore, the sample size will be approximately 2800 women who are negative for intraepithelial lesions or malignancy.

## Discussion

Previous studies have confirmed the prophylactic effect of HPV vaccination on cervical cancer in females aged 9–26 years. The FUTURE study showed that the quadrivalent HPV6/11/16/18 vaccine reduced the incidences of HPV16- and HPV18-related cervical precancers and cervical cancer in women aged 16–26 years [10]. The study, which investigated the cost-effectiveness of the vaccine worldwide, recommended the first target age for vaccination to be 9–14 years and the next target age to be up to 26 years [10]. There have been only two large studies on the effects of HPV vaccination in adult women. One of the two was the VIVIANE study using the AS04-HPV-16/18 vaccine, which indicated that the vaccine is approximately 80% effective against persistent infection in adult women [5]. The other study was a randomized controlled trial that demonstrated the efficacy of the quadrivalent HPV6/11/16/18 vaccine in reducing the rate of persistent infections among adult women [9]. Therefore, generating new evidence about HPV vaccination in the young adult population will be worthwhile, particularly in the postlaunch real-world setting.

As mentioned above, adult women are not covered under the national immunization program of Japan due to cost-effectiveness concerns related to high HPV16/18 prevalence. In this study, we will enroll only women with normal cytology results because our previous study (FCCS study) revealed that women from Fukui Prefecture with normal cytology results were basically negative for HPV16/18 [7].

As the Japanese cervical cancer screening program uses only cytology without HPV-DNA testing, there are no available data on the detection of HPV16/18 DNA. However, the FCCS study has shown that the HPV16/18 infection rates were significantly lower among those with normal cytology results than among those with abnormal cytology results (1.0% vs. 22.0%). Therefore, the present study will target women with normal cytology results [7].

While young adult women aged 27–45 years are not covered under the national immunization program, they have the chance to visit gynecologists periodically to undergo cervical cancer screening provided through the national program. This regular visit is a suitable opportunity for vaccine recommendation, as normal cytology results still indicate susceptibility to HPV infection, and during the visit, the necessity of vaccination can be conveyed by the physician directly to the patient, not to her parents or guardians.

A possible limitation of the study is that the study design involves a nonrandomized, and not randomized, intervention. If the study were to adopt a randomized intervention design, we speculate that the participation of the patients cannot be assured under current circumstances in Japan, where the MHLW has suspended its strong recommendation for HPV vaccination.

The findings of this trial might provide robust local evidence supporting HPV vaccination among young adult women aged 27–45 years who show negative cytology results. By utilizing the data from this study, we intend to develop a platform covering both cervical cancer screening and HPV vaccine recommendations for those proven to have negative cytology results. This new platform might serve as an effective means to drive HPV vaccination among those not covered under the national immunization program of Japan and raise questions about the rigid state of the 5-year national immunization program. This study could provide local supportive evidence for preventing HPV infection through HPV vaccination in young adult women and thus contribute to public health improvement in Japan, where active recommendation of HPV vaccination has been suspended by the MHLW for a long time.

## Data Availability

This trial is registered in the Japan Registry of Clinical Trials (jRCTs051190039) and on ClinicalTrials.gov (NCT04022148). https://jrct.niph.go.jp/latest-detail/jRCTs051190039 https://clinicaltrials.gov/ct2/show/NCT04022148

## Abbreviations

HPV: Human papillomavirus
MHLW: Ministry of Health, Labour and Welfare
FCCS study: Fukui Cervical Cancer Screening study
